# Biochar and Manure Applications Differentially Altered the Class 1 Integrons, Antimicrobial Resistance, and Gene Cassettes Diversity in Paddy Soils

**DOI:** 10.3389/fmicb.2022.943880

**Published:** 2022-06-29

**Authors:** Niyaz Ali, Yinfu Lin, Ligeng Jiang, Izhar Ali, Ishtiaq Ahmed, Kashif Akhtar, Bing He, Ronghui Wen

**Affiliations:** ^1^State Key Laboratory for Conservation and Utilization of Subtropical Agro-Bio-Resources, College of Life Science and Technology, Guangxi University, Nanning, China; ^2^Guangxi Key Laboratory of Agro-Environment and Agric-Products Safety, College of Agriculture, Guangxi University, Nanning, China; ^3^Department of Regional Science Operations, La Trobe Rural Health School, Albury-Wodonga, VIC, Australia

**Keywords:** antibiotic resistance, biochar, environment, gene cassettes, integrons, manure

## Abstract

Integrons are genetic components that are critically involved in bacterial evolution and antimicrobial resistance by assisting in the propagation and expression of gene cassettes. In recent decades, biochar has been introduced as a fertilizer to enhance physiochemical properties and crop yield of soil, while manure has been used as a fertilizer for centuries. The current study aimed to investigate the impact of biochar, manure, and a combination of biochar and manure on integrons, their gene cassettes, and relative antimicrobial resistance in paddy soil. Field experiments revealed class 1 (CL1) integrons were prevalent in all samples, with higher concentration and abundance in manure-treated plots than in biochar-treated ones. The gene cassette arrays in the paddy featured a broad pool of cassettes with a total of 35% novel gene cassettes. A majority of gene cassettes encoded resistance to aminoglycosides, heat shock protein, heavy metals, pilus secretory proteins, and twin-arginine translocases (Tat), TatA, TatB, and TatC. Both in combination and solo treatments, the diversity of gene cassettes was increased in the manure-enriched soil, however, biochar reduced the gene cassettes’ diversity and their cassettes array. Manure considerably enhanced CL1 integrons abundance and antimicrobial resistance, whereas biochar amendments significantly reduced integrons and antimicrobial resistance. The results highlighted the differential effects of biochar and manure on integrons and its gene cassette arrays, showing increased abundance of integrons and antibiotic resistance upon manure application and decrease of the same with biochar. The use of biochar alone or in combination with manure could be a beneficial alternative to mitigate the spread of antimicrobial resistance and bacterial evolution in the environment, specifically in paddy soils.

## Introduction

Excessive use of antibiotics and their environmental contamination have ecological and evolutionary consequences, including antimicrobial development. Antibiotic resistance evolves as a result of mutations in the genome, with horizontal gene transfer (HGT) being a well-known phenomenon in this context (Zhu et al., 2017; Lima et al., 2020). HGT facilitates bacterial adaptive behavior and rapid evolution and promotes antimicrobial resistance genes (ARGs) across bacteria species in the environment (Martínez, 2008; Zhu et al., 2017; Ali et al., 2020a). Mobile genetic elements (MGEs) mediate the HGT, which could enhance the lateral gene flow among the clinical isolates (Stecher et al., 2012; Chen et al., 2016; An et al., 2018; Khan et al., 2022). Thus, understanding the HGT is critical for resolving health issues caused by antimicrobial resistance and their subsequent dissemination in the environment.

Integron is a gene acquisition system that resides in the genome of bacteria and is prevalent in Gram-negative organisms. Integrons play a vital role in bacterial evolution and antimicrobial resistance (AMR) development, as they are involved in the propagation and expression of gene cassettes (Liebert et al., 1999; Michael et al., 2004; Frost et al., 2005; Wozniak and Waldor, 2010; Ghaly et al., 2020). Integrons are made up of two basic components: a structural component and a gene cassette array. The initial component is at the 5′ conserved region (5′CS), which includes an integrase (intI) gene, an attI recombination site, and a promoter (Pc) region. This platform acquires GCs and expresses them in a row if the gene cassette lacks the open reading frames (ORF; Peterson and Kaur, 2018; Ali et al., 2020a; Ghaly et al., 2020).

The gene cassettes may consist of one or more open reading frames (ORFs) and recombination cassettes joining the attC site. Integron integrase enzyme, encoded by intI gene, is a site-specific tyrosine recombinase enzyme that has the specialized activity of integrating and excising gene cassettes, aiding the acquisition of genetic information (Gillings et al., 2009; Yang et al., 2021). The expression of integrase genes can be induced by bacterial conjugation, transformation, starvation, antimicrobial resistance, and environmental stress (Cambray et al., 2011; Bellanger et al., 2014; Amarasiri et al., 2020). Acquisition of GCs by integrons and their subsequent expression led to bacterial evolution and the development of adaptive antibiotic resistance (Hall and Collis, 1998; Berendonk et al., 2015; Yang et al., 2021).

Rice (*Oryza sativa* L.) has a long plantation history and serves as a staple food for the growing population across the globe. Various fertilizers are used to boost the output and quality of the rice crop. Among the fertilizers, manure is one of the oldest and most common organic fertilizers (Iqbal et al., 2019; Ali et al., 2022). Manure, as compared to fertilizers, is a major source of antibiotics and antimicrobial resistance genes due to the undefined use of antibiotics in animal farming (Lin et al., 2016; Rahman et al., 2018). Different antibiotics and antibiotic resistance genes have been identified in the rice rhizosphere (Uddin et al., 2020). The abundance of ARGs in paddy is positively correlated with antibiotics, soil properties including pH and soil organic matter (SOM), and heavy metals such as As, Cd, Cu, and Zn (Tang et al., 2015; Lin et al., 2016; Cao et al., 2020; Zhao et al., 2020).

In recent decades, biochar amendment has been introduced to improve soil quality and agricultural productivity as well as to reduce the antimicrobial resistance in the soil (Duan et al., 2017; An et al., 2018; Li et al., 2020). According to Gul et al. (2015), beneficial changes in soil physiochemical parameters are dependent on biochar characteristics such as its source and production, temperature, and native characteristics of the soil, including its texture. In an anaerobic digestion system, adding biochar to cattle slurry and wastewater could lower the environmental dissemination of ARGs (Sun et al., 2018), and the addition of the same to pig manure could effectively reduce the bio-availability of the antibiotics (Ngigi et al., 2020).

Direct conversion of manure into biochar is an alternative approach to reduce the spread of antibiotic resistance genes from manure to the field environment (Zhou et al., 2019). However, according to certain studies, the effect of biochar ARGs depends on the type of biochar and manure utilized (Cui et al., 2017). The abundance of integrons and accompanying antimicrobial resistance in paddy fields in a typical agricultural ecological setting of Guangxi was substantially higher than in sugarcane fields, according to our earlier study (Ali et al., 2020a). The paddy fields have different forms of anthropogenic inputs resulting in the high abundance of integrons and their subsequent gene cassettes array. The current study was set to investigate the differential effects of biochar, manure, and their combined application on integrons, antimicrobial resistance, gene cassettes diversity, and their subsequent impact on soil physiochemical properties.

## Materials and Methods

### Experimental Design and Sample Collection

The field experiments were conducted in the experimental rice fields of Guangxi University in the first growing season of rice (usually March to the middle of July in southern China), with a randomized complete block (RCB) design, having a plot size of 3.9 × 6 m (23 m^2^) in three replicates for each. The field experiment was categorized into four treatments, i.e., control (untreated) ©, biochar (B), application (20 t ha^−1^), manure, amendment (15 t ha^−1^) (M), and combination of biochar and manure (10 + 7.5 t ha^−1^) (BM) for each treatment. The targeted field crop was the noodle rice “Zhenguiai” and all the plots received the same basic agronomic procedures, such as irrigation and pesticide application, throughout the growing season. Due to the slow decomposition of manure and biochar, both were applied 25 days before transplantation, so the nutrient could be available for the plant. At the physiological maturity stage, when plant growth was at its peak, soil samples were taken near the rhizosphere and were transported to the lab in an icebox and stored at −80°C for further analysis.

### Soil, Biochar, and Manure Physiochemical Characteristics

From each treatment, three randomly replicated soil samples were selected and the bulk density (BD) was assessed by the core method as previously described by Grossman and Reinsch (2002). The obtained BD was then used to measure the total porosity of soil using the following equation:

(1) $$\mathrm{Porosity}=\left(1-\frac{BD}{PS}\right)\times 100$$

Soil moisture content (SMC) was determined as per Ledieu et al. (1986) and soil pH was measured after shaking it with distilled water at a 1:2.5 (w/v) solid-to-water ratio for 1 h. The soil pH values were evaluated using a digital pH meter (Thunderbolt PHS-3C China). Soil organic carbon (SOC) was assessed *via* the oxidation method described previously by Cambardella et al. (2001) and Bao (2000). Then the soil samples (0.5 g) were digested with 5 ml of 1 mol K_2_Cr_2_O_7_ and 5 ml of concentrated H_2_SO_4_, boiled at 175°C for 5 min, and titrated with FeSO_4_ (Bao, 2000). For soil N content, 200 mg of soil was digested using salicylic acid and sulfuric acid hydrogen peroxide as per the procedure of Ohyama et al. (2004) and then the total N was analyzed by the micro-Kjeldahl method (Jackson et al., 1995). The available concentration of P and K were calculated according to Murphy and Riley (1962) and Pierzynski (2000), respectively. The biochar used in this experiment was made from cassava straw carbon burnt in a traditional kiln by following the procedure reported previously (Ali et al., 2022).

The physiochemical properties of soil, manure, and biochar before the experiment are presented in Table 1.

**Table 1:** Physical and chemical characteristics of soil, manure, and biochar before the experimentation.

Properties	Soil	Manure	Biochar
Porosity (%)	40.12	-	-
Moisture content (%)	11.23	-	-
Bulk density (g cm^−3^)	1.38	0.81	0.78
pH (water)	5.94	7.75	8.8
Total C (g kg^−1^)	-	-	674
S (g kg^−1^)	-	-	2.39
H (g kg^−1^)	-	-	3.81
SOC (g kg^−1^)	13.06	146.33	-
SOM (g kg^−1^)	23.4	254.63	-
Total N (g kg^−1^)	1.34	9.80	5.43
Total P (g kg^−1^)	0.62	10.12	46.33
Total K (g kg^−1^)	-	14.22	48.33
Available N (mg kg^−1^)	130.71	-	-
Available P (mg kg^−1^)	22.21	-	-
Available K (mg kg^−1^)	230.50	-	-
C:N ratio	7.16	14.92	124.12

*Total C, total carbon; SOC, soil organic carbon; SOM, soil organic matter; N, nitrogen; P, phosphorous; K, potassium; C:N, carbon-to-nitrogen ratio; S: sulfur; and H, hydrogen*.

### DNA Extraction

The soil DNA extraction kit (QIAGEN Korea) was used for extracting DNA, according to the instructions of the manufacturer, the concentration of DNA was quantified using Nano-Drop (model One C), and the samples were preserved at −80°C for further proceedings.

### Quantitative PCR

To quantify the abundance of Class1 integron integrase gene and 16S rRNA genes, the qPCR was conducted and the conditions were set as reported previously by Barraud et al. (2013) and An et al. (2018). SYBR® Premix Ex Taq™ II (Takara, Japan) was used for conducting qPCR using the Roche real-time PCR (model Light Cycler 480II) system. The samples were replicated in triplicate using the conditions set previously (Stalder et al., 2014; Chen et al., 2016). A standard curve with the corresponding gene fragments was created using the diluted plasmid. The relative abundance of integron-integrase genes was calculated by dividing the 16S rRNA gene copy number by that of the integrase gene copy number.

### Gene Cassette Libraries

Class 1 integron variable regions were amplified, using the primer pairs suggested previously (Stalder et al., 2014), in a 50-μl reaction mixture containing 20 ng of DNA, 25 μl of 2× premix Takara Ex Tag (Takara Japan), and 2.5 μl of each primer in PCR grade water. The PCR conditions were optimized as follows: an initial step at 95°C for 3 min, followed by 35 cycles at 94°C for 30 s, 55°C for 30 s, 72°C for 2 min and 30 s, and a final step at 72°C for 10 min (Stalder et al., 2014; Bengtsson-Palme et al., 2018). Each DNA sample was amplified in triplicate, and the amplicons were pooled and purified using a gel purification kit (Vazyme, China). The PCR-purified amplicons were quantified and ligated into the M13 vector and then transferred to competent cells of *E. coli* DH_5α_ following the manufacturer’s instructions (Vazyme, China).

Seventy-two clones were selected randomly in triplicate from each sample, and Sanger was sequenced after confirmation of a gene cassette size of 153 bp (153 bp indicates empty cassette array) by amplifying with M13 reverse and forward primers followed by sequencing. To avoid ambiguous bases, the sequences were confirmed after assembling the primers, and gene cassettes were analyzed to see if the sequences contained the two primer pairs (3′CS and 5′CS). Putative attC sites were manually searched out and the attC sites were further confirmed manually and then bio-informatically as previously described by Cury et al. (2016) and Pereira et al. (2020). The sequence annotation was performed by BLASTX against NCBI non-redundant protein database with a threshold e-value of 1 × 10^−5^. A potential gene was annotated with sequence identity ≥35% and an alignment length of ≥30 amino acids. The sequences were further annotated with BLASTN and BLASTX of NCBI. Those sequences annotated as hypothetical, with no hit in NCBI, were further authenticated using the NCBI ORF finder.^1^

### Statistical Analysis

One-way ANOVA was performed using the SPSS V20.0 (SPSS Inc., Chicago, Ill., United States) and Statistix 8.0 (Analytical Software 2105 Miller Landing Rd. Tallahassee, FL 32312) for data analysis, and figures were plotted using Sigma Plot 12.5 software and Origin 9.1. Means of all replicates were compared using the least significant difference test at the probability level of 0.05.

## Results

### Physicochemical Properties of the Soil

Physicochemical properties of the soil, including moisture content (%), bulk density (g cm^−3^), porosity (%), pH (1:2.5 (w/v) solid-to-water ratio), SOC, total nitrogen (TN), available nitrogen (AN), available phosphorous (AP), and available potassium (AK), were significantly affected by the application of biochar (B), manure (M), and combination of biochar and manure (BM; Table 2). Compared to the control, M, BM, and B treatment significantly increased soil moisture by 29%, 14%, and 20%, porosity by 61%, 4%, and 32%, SOC by 62%, 24%, and 36%, and pH by 18%, 6%, and 12%, respectively.

**Table 2:** Impact of biochar manure and their combined application on soil physicochemical properties.

Soil physicochemical properties	Control	Biochar (B)	Manure (M)	BM
Moisture content (%)	11.2b	14.51a	13.25a	13.88a
Bulk density (g cm^−3^)	1.37a	1.3b	1.29b	1.295b
Porosity (%)	40.21c	64.89a	43.27c	54.08b
pH (water)	5.92c	7.04a	6.36b	6.7b
SOC (g kg^−1^)	9.61c	15.61a	13.46a	14.535a
TN (g kg^−1^)	1.29d	1.62c	1.73b	1.83a
AN (mg kg^−1^)	128.3b	160.3a	165.4a	162.85a
AP (mg kg^−1^)	21.96c	25.32b	27.22a	27.77a
AK (mg kg^−1^)	240.53b	253.94	348.2a	329.07a

*SOC, soil organic carbon; TN, total nitrogen; A, available nitrogen; AP, available phosphorous; and AK, available potassium. Values followed by the same letters, within column, are not significantly different, with *p* ≤ 0.05. The statistical letters show the difference across different treatments*.

However, AN and AK levels were more enhanced by 28% and 44% in the manure-treated plots than in the control, after BM and B treatment, respectively. Similarly, TN and AP were significantly (34% and 26%, respectively) higher in the BM application than in the control treatment, whereas bulk density was 5%, 6%, and 5% lower after B, M, and BM treatments, compared to that in the control. Therefore, manure and biochar were both concluded to have distinct effects on the physicochemical properties of the soil.

### Integrons and 16S rRNA Abundance

**Figure 1:**
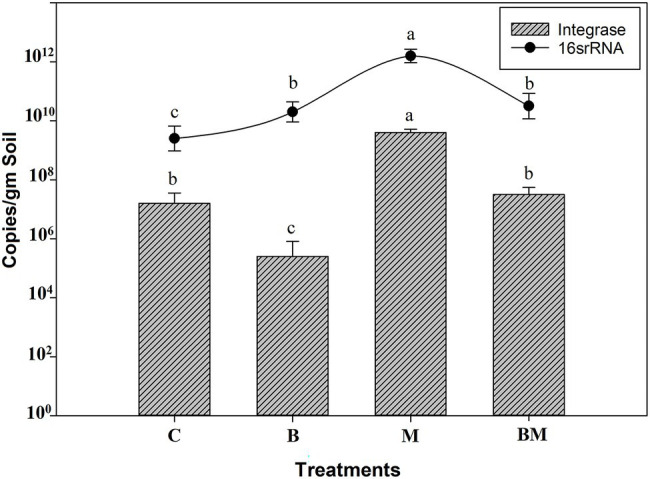
Effect of biochar (B), manure (M), and their combined application (BM) on concentration/copy number of the CL1 integron-integrase gene and 16S rRNA. The columns represent integrase, and the curve indicates 16S rRNA copy number per gram of soil in each treatment. The bars above the columns and the curve represent the SE across the replicates. Differences in copy numbers of each sample group were evaluated using the least significant difference (LSD) method. Different letters (a, b, and c) above the columns and curve indicate statistical significance at *p* < 0.05.

In the manure-amended soil, the prevalence and relative abundance of integrons were higher than in the control (*p* < 0.05). While biochar application reduced the abundance of integrons in the soil, it did so at a lower level than untreated soil (*p* < 0.05). The prevalence and relative abundance of integrons were higher in the manure-amended soil than in the control (*p* < 0.05). While the application of biochar significantly reduced the abundance of integrons in the soil (*p* < 0.05), the level was lower than in untreated soil (Figure 1). The combined application of manure and biochar showed the addition of biochar significantly (*p* < 0.05) reduced the abundance of integrons in the BM-amended soil, the abundance being almost equal to that in the untreated soil. It was less than that in the manure-amended soil and elevated than in biochar-treated soil. Results showed biochar amendment affects the integrons carried by the bacterial population in the paddy soil environment and significantly mitigates their abundance.

### Gene Cassette Clone Library

**Figure 2:**
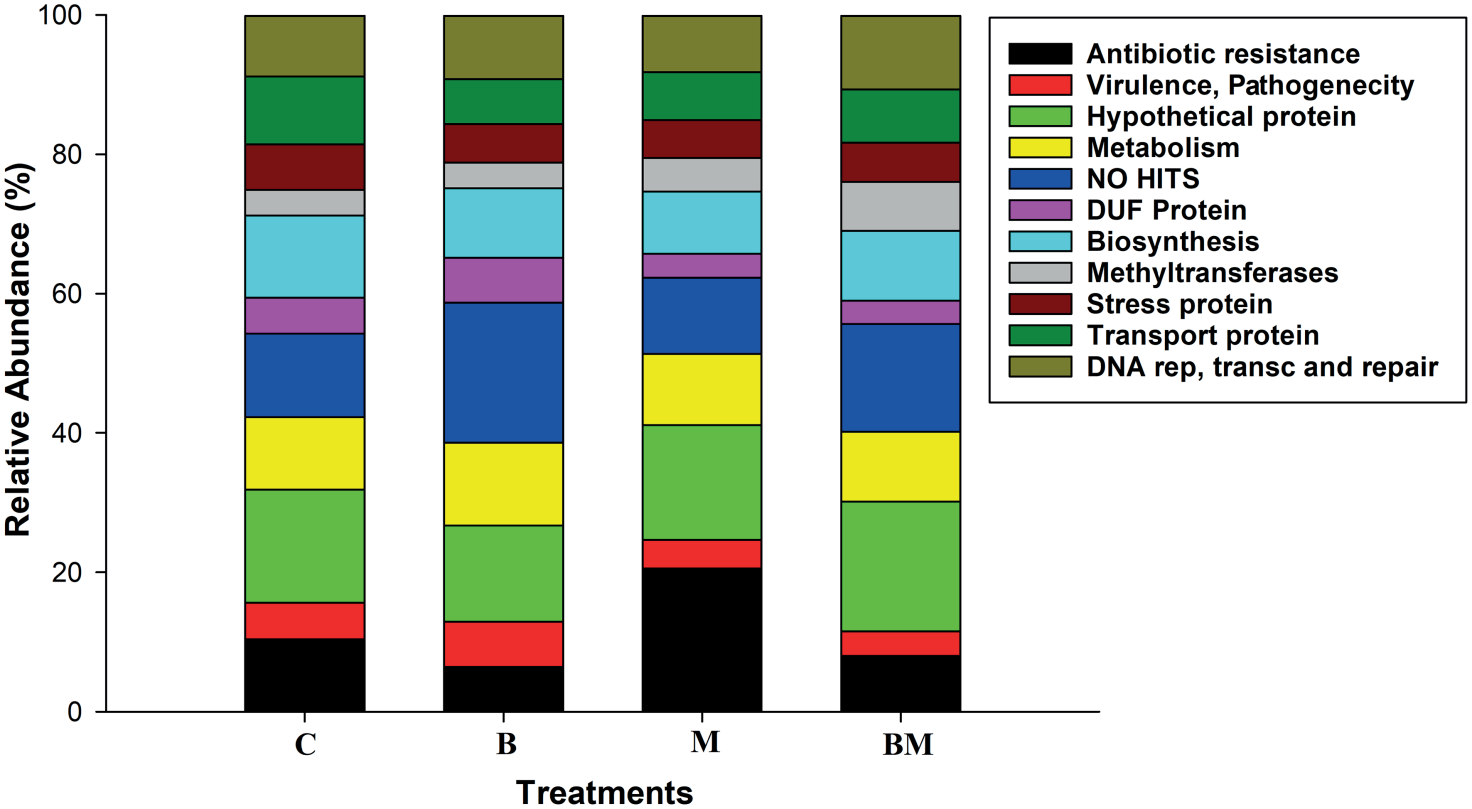
Changes in relative abundance of various proteins in response to biochar, manure, and their combined application in the paddy field. C, control; B, biochar; M, manure; and BM, biochar + manure. Different colors in the column indicate different types of proteins, as listed in the index box.

The impacts of manure and biochar application on integrons were further investigated by clone library analysis. The most relevant GCs were merged. We found approximately 35.3% of gene cassettes to be empty, especially in the biochar-alone-amended (B) and when in combination with manure (BM); this value was <35% in manure-amended soil (M) and untreated soils (C). Approximately 20% of gene cassettes showed no-hit in NCBI in the biochar-amended soil, and 6.2% of gene cassettes were transcribed into ARGs while 13.5% were hypothetical proteins in the biochar-amended soil (Figure 2). Approximately 35% of the gene cassettes in this study were novel, either showing no-hit in the NCBI database or having very low e values in amino acid identity.

The manure-amended soil carried approximately 21% ARG cassettes, which was higher than that in the untreated (10.3%) and biochar-amended soils (6.2%). Hypothetical proteins accounted for more than 16%, and gene cassettes showing no-hit were 10.5% in the manure-amended soil. The untreated soil samples carried 10.3% ARG cassettes, and 7% of the gene cassettes showed no-hit in the NCBI database while 16% were transcribed into hypothetical proteins (Figure 2). In addition, we found other gene cassettes with various biological functions, like stress response, mobility, biosynthesis, cell synthesis, DNA replication, transportation, methylation, and DNA repair.

**Figure 3:**
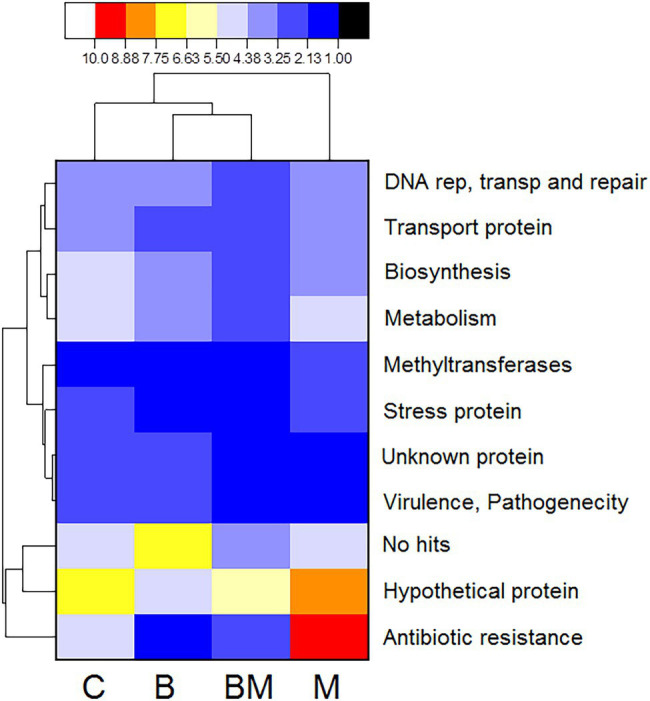
Heat map analysis of the frequency of proteins translated from various gene cassettes after C (untreated), B (biochar), BM (biochar + manure), and M (manure) treatments. Data were analyzed for the presence of multiple proteins from the gene cassettes based on the percentage of gene coverage as a measure of relative gene frequency in each sample. Each row of the heat map represents an individual gene cassette protein and the column represents different treatments. The color gradient key displays a linear scale of the percent gene coverage as a measure of relative gene frequency. The red color shows high frequency and black shows the lowest.

The cassettes composition data for each treatment level were clustered according to the abundance distribution and the degree of similarity of those sequences. According to the clustering results, the cassettes and the treatments were ranked separately and presented through heat maps. Color gradients were used to distinguish the high and low abundance of the cassettes (Figure 3). In the experiment group treated with manure, the frequency of ARG in those gene cassettes is the highest; the detection frequency of ARG in the biochar treatment group was the lowest. In contrast, the frequency of unknown sequences in those gene cassettes increased in the biochar experimental treatment group (Figure 3).

Gene cassettes transcribed into twin-arginine protein were found frequently in all samples. Stress-related gene cassettes that were translated into heat shock protein of different types and functions were also identified. The other gene cassettes, which are responsible for the pilus secretions system, were frequently detected in these samples. Some gene cassettes were responsible for the production of energy, and we also found gene cassettes resistant to heavy metals.

### Effects of Biochar and Manure on the Diversity of Gene Cassettes

**Figure 4:**
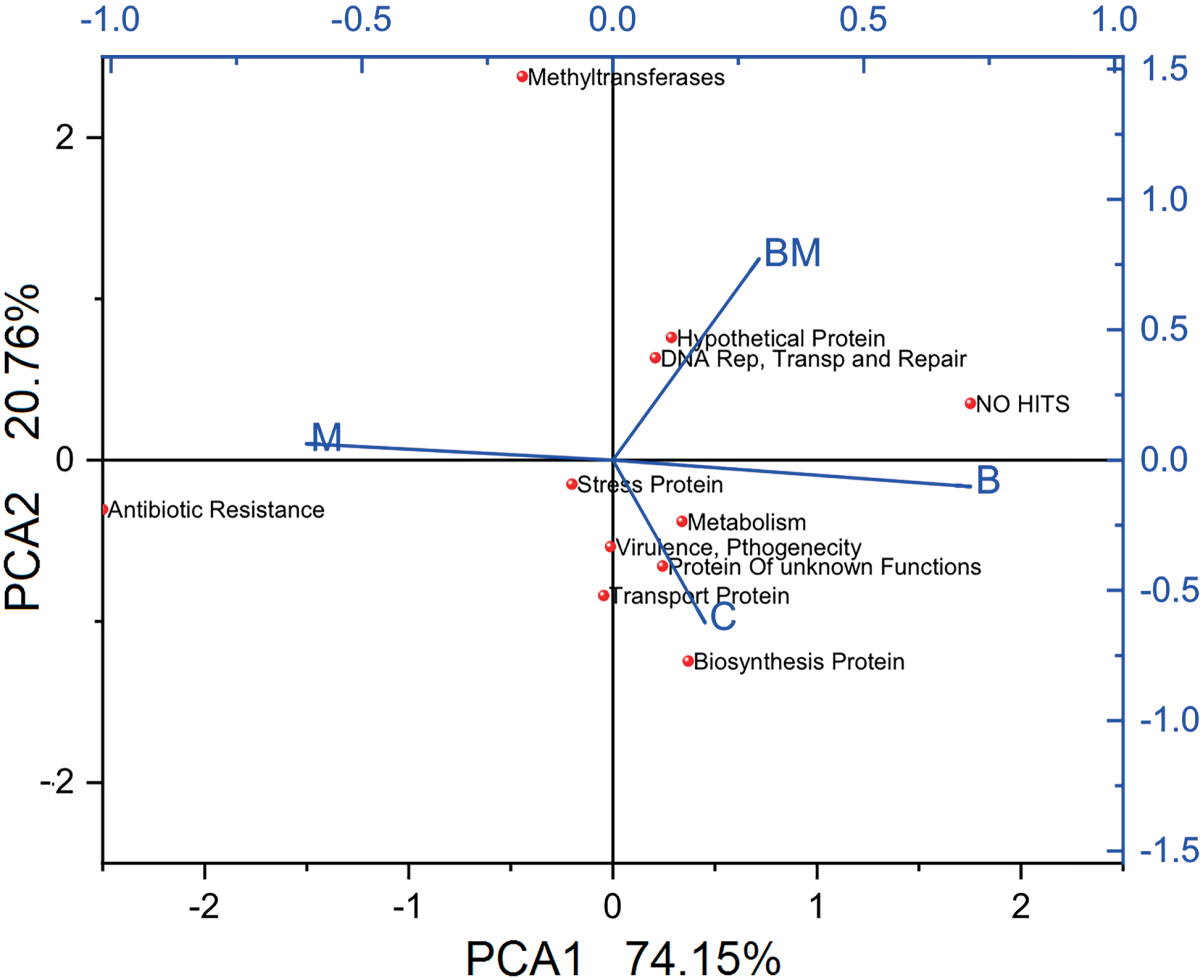
Principal coordinates analysis of gene cassettes. Different gene cassettes from each treatment were analyzed *via* principal coordinates analysis using the Bray–Curtis distance measure, and clustered using the PAM algorithm (Woolley and Athalye, 1986). C, untreated; B, biochar; BM, biochar + manure; and M, manure.

A total of 864 clones were prepared, 216 from each treatment, in triplicates of 72. The gene cassettes having close functions were merged and presented in Figure 4. Analysis of the sequences showed the manure amendment significantly (*p* < 0.05) increased the diversity of gene cassettes. While a large number of gene cassettes harbored antibiotic resistance of diverse types (*p* < 0.05) in the manure-amended soil, the average number of ARGs in the manure-treated soil was 13. The lowest numbers were found in the biochar-amended soil, with only two ARGs, and an almost close average number 2.33ARGs were observed in the manure-biochar combination-amended soil. The untreated soil (C) samples contained more ARG cassettes than both B and BM samples and the average number was 4.5 for each sample.

Stress-resistance gene cassettes were found in abundance in the untreated samples; they were significantly more (*p* < 0.05) in biochar-treated soil, although with no-hit in the NCBI database. The PCA1 showed a variation of 74.15% while PCA2 was 20.76%, implying that manure treatment is more distinct compared to others, and control and biochar treatment are more closely related to each other (Figure 4). The results collectively revealed that BM is more distant from M and closer to B; PCA confirmed the biochar effects on gene cassettes to be more prominent, resulting in closeness to the control and minimization of the impact of manure on gene cassettes.

**Figure 5:**
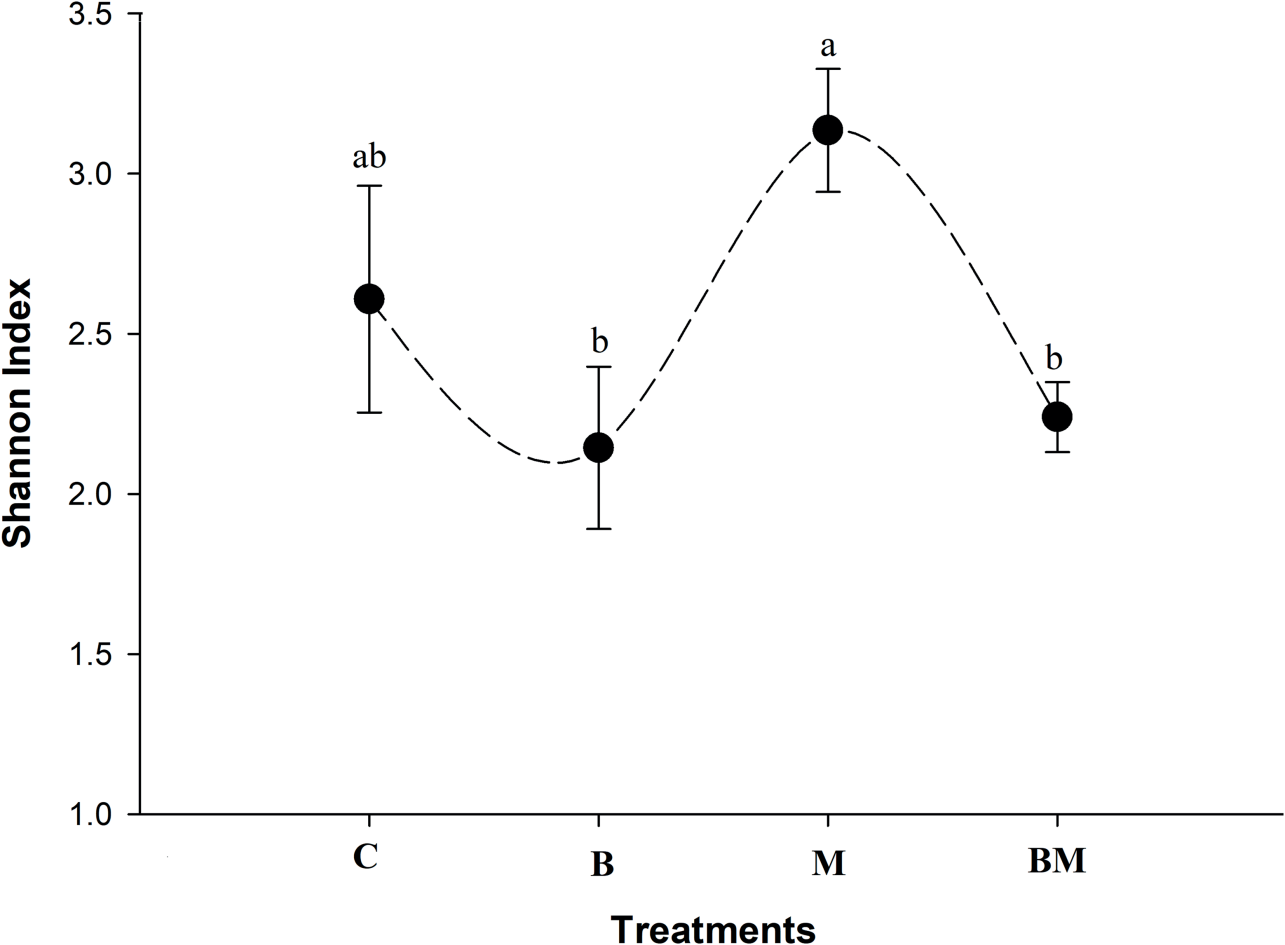
Alpha diversity of gene cassettes revealed by Shannon index of the different treatments. Biochar amendment significantly decreased the diversity of gene cassettes in paddy soil, and the same happened when biochar was combined with manure (a and b above the curve represent statistical difference at *p* < 0.05). Application of manure elevated the diversity of gene cassettes. Different letters above the line indicate statistical significance at the *p* < 0.05.

Overall, diversity across samples was highest in the manure-amended soil (Figure 5). The lowest number of gene cassettes was found in the biochar-amended soil samples (*p* < 0.05), and the second-diverse group of gene cassettes was found in the untreated samples. In terms of no-hit in the NCBI database, biochar-treated soil contained a higher number and diverse range of gene cassettes. Shannon index showed manure amendment to increase the diversity of gene cassettes in the soil, while the addition of biochar significantly decreased (*p* < 0.05) the gene cassettes’ diversity.

### Novel Gene Cassettes

Manure and biochar treatment showed different effects on integron gene cassettes. Although integrons are mostly related to antibiotic resistance, we found some novel gene cassettes as well, with different functions. One cassette that encodes HlyD family efflux transporter periplasmic protein shared 43% amino acid identity with hemerythrin HHE cation binding domain protein and played a major role in multidrug resistance (Kim et al., 2016).

**Figure 6:**
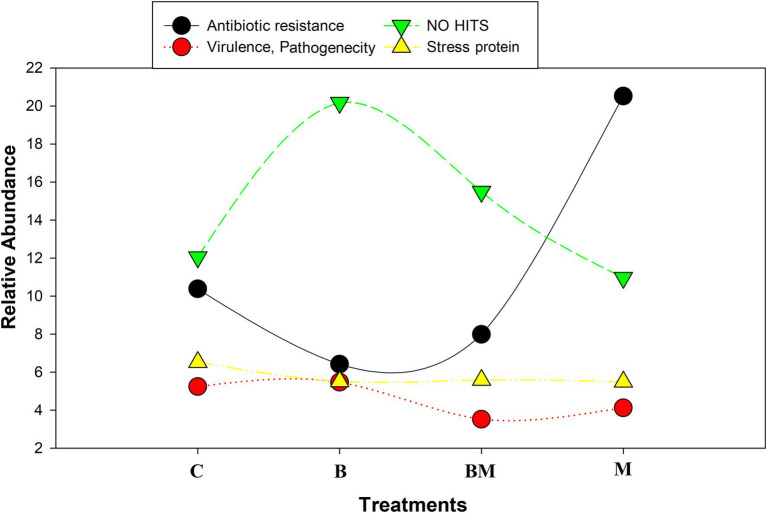
Representative four scattered lines. The black line represents antibiotic abundance in all treatments; the green line represents gene cassettes that show no hit in NCBI database; the yellow line and stacks represent the virulence and pathogenicity related to gene cassettes; the red line represents stress-related gene cassettes. The antibiotic resistance genes are significantly decreased (*p* < 0.05) with biochar application in combined as well as sole treatments. Biochar amendment significantly changes the gene cassette arrays and novel gene cassettes are incorporated into the array (*p* < 0.05).

We found gene cassette encoding drug resistance that shared only 65% of amino acid identity with ABC-type multidrug transport system, permease component (Orelle et al., 2019; Li et al., 2020). Another gene cassette shared 75% amino acid identity with twin-arginine translocase subunit TatC, and increased the virulence of some bacterial species (Berks et al., 2014). We found some gene cassettes to show no-hit in the database at all. Their further analysis (Figure 6) and ORF readings represented diverse functions, similar to RNA-binding protein, holiday junction proteins, and DNA-methylation protein (Ghodge and Raushel, 2018).

A gene cassette transcribed into the vicinal oxygen chelate (VOC) family had beta-alpha-beta motifs that showed 74% amino acid identity in the database and provided metal coordination with the environment. The VOC is found in a variety of metal proteins, including type I extra diol dioxygenases, glyoxalase I, and a group of antibiotic resistance proteins, and performs a key role in the degradation of aromatic compounds. We found novel gene cassettes having the properties of metallophosphatases, despite showing only 59% amino acid identity in a database. A gene cassette showed 72% amino acid identity with NikR, and was a transcription factor that regulates nickel uptake (Zeng and Zamble, 2017).

We found some gene cassettes related to the type IV secretion system, type II system, and their sub-units that secrete proteins, sharing a homology of 73% within the database, and 43% homology with the N terminal region of pilus secretary protein. These secretion systems are responsible for motility, attachment, colonization, and horizontal gene transfer (Sgro et al., 2018).

## Discussion

### Physicochemical Properties of the Soil

In recent years, biochar has been introduced as a fertilizer to improve soil quality and crop yield (Duan et al., 2017; An et al., 2018; Ali et al., 2020b; Li et al., 2020; Ullah et al., 2021). Addition of biochar into paddy soil enhanced physicochemical properties, i.e., moisture content, porosity, SOC, and pH, compared to that in the control (Table 2). In pyrolysis, plant biomass does not lose much of the naturally available nutrients (Ali et al., 2020b). The addition of manure to paddy soils increased the available nitrogen (AN) and potassium (AK) levels. Combined treatment with biochar and manure enhanced the total nitrogen (TN) and available phosphorous (AP) levels compared to non-biochar and manure treatments (C). These changes in manure-treated paddy fields were due to the considerable number of macronutrients and micronutrients supplied by manure. The positive effects of biochar and manure in combination with physiochemical properties of soil were similar to those reported recently by Iqbal et al. (2019) and Ali et al. (2020b).

Positive impacts of biochar amendment might be due to the presence of different organic and inorganic forms of mineral elements, however, the concentration of these nutrients varies with the source and production temperature of biochar (Gul and Whalen, 2016; Khan et al., 2020; Ullah et al., 2021). Furthermore, the addition of organic manure in combination with biochar allows more nutrients to the soil due to the availability of numerous nutrients in organic manure (Iqbal et al., 2020) and biochar (Ali et al., 2021). Our results showed that biochar in combination with organic manure improved soil fertility as compared to sole biochar or sole organic manure-treated soil.

### Abundance of Class 1 Integrons and 16S rRNA

This study showed that the addition of manure could efficiently elevate the integrons in the paddy environment, hence confirming manure as a key source of integrons and ARGs in the environment. Antibiotics contained in manure could also provide selective pressure for microbes and lead to increased integrons in the soil. Previous studies had also shown manure as a viable source of integrons and antimicrobial resistance owing to the extensive use of antibiotics in livestock (Lima et al., 2020; Wang et al., 2020). The concentration of integron-integrase gene in the biochar-amended paddy was lower than that in manure-amended paddy (M) and the control (C), thereby suggesting that biochar has negative effects on the concentration of integrons. The main reason why biochar addition alters microbial community is that it could induce changes in nutrient cycles, plant growth, and soil organic matter. That could lead to a decrease in integron abundance (Ameloot et al., 2013; Anyika et al., 2015). The combination of B with M significantly attenuated the increasing effects of manure on integrons and antimicrobial resistance.

### Gene Cassette Clone Library

Gene cassettes are enormous reservoirs of genetic diversity and play a major role in the evolution of bacteria to specific conditions (Gillings, 2014). Our clone library analysis of gene cassettes showed that most of the gene cassettes encoding polypeptides either had no homology in the database or had so with hypothetical proteins and were novel, which was in line with the previous studies conducted in the environmental settings of integron gene cassettes (Elsaied et al., 2007; Koenig et al., 2009; An et al., 2018; Ali et al., 2020a).

Biochar application reduced the gene cassette diversity, though biochar-amended paddy soils had more novel gene cassettes, hence confirming biochar has distinct effects on integrons and their gene cassette arrays, and integrons have evolved their cassette arrays owing to biochar amendment pressure. Integrons have been continually evolving their cassette arrays and acquiring new gene cassettes due to selective pressure (Gillings, 2014; An et al., 2018).

Biochar has carbon aromatic compounds that play a role in changing bacterial composition (Ameloot et al., 2013; Anyika et al., 2015); we found a gene cassette that showed 74% amino acid with the VOC family (Mikkonen et al., 2018), and provided metal coordination with the environment. The VOC is found in a variety of metal proteins, including type I extra diol dioxygenases, glyoxalase I, and a group of antibiotic resistance proteins, and plays a key role in the degradation of aromatic compounds (Milanowski et al., 2019). Biochar significantly reduced the ARG cassettes upon treatment alone or in combination with manure, thus indicating its negative impact on the resistance genes upon sole or combined application. Therefore, biochar could serve as an approach to reducing the dissemination of antibiotic resistance in the environment (Ngigi et al., 2020).

### Antibiotic Resistance Genes

The majority of the gene cassettes implicated in multi-drug resistance were found in M and C treatments. We also found different classes of ARGs in M and C treatments, separately; however, the most prevalent were the aminoglycosides across all treatments. They harbored nucleotide transferase enzyme, resisted different types of aminoglycosides, and had a resemblance with the kanamycin resistance gene (Martí et al., 2019; Ali et al., 2020a).

### Novel Gene Cassettes

We observed some novel gene cassettes that had different functions, for example, encoding HlyD family efflux transporter periplasmic protein and sharing 43% amino acid identity in the database (Kim et al., 2016). Another gene cassette for hemerythrin HHE cation binding domain protein was found that protects the anaerobic and microaerophilic organisms against oxidative damage, and supplied oxygen to particular enzymes and pathways in aerobic and facultative species. This is one of the evolutionary advantages of bacteria since oxygen availability in the paddy soils is scarce and the integron gene cassette arrays incorporate the relevant gene cassette to fulfill the requirements (Alvarez-Carreño et al., 2016). Gene cassettes encoding drug resistance shared only 65% amino acid identity with the permease component of the ABC-type multidrug transport system, (Orelle et al., 2019; Li et al., 2020). Another gene cassette, which shared 75% amino acid identity, was transcribed into twin-arginine translocase subunit TatC, which can increase the virulence of some bacterial species (De Buck et al., 2008).

We also noted that several gene cassettes gave no-hit in the database, although further analysis and ORF readings represented diverse functions, as in RNA-binding protein, holiday junction proteins, and DNA methylation protein. We detected previously unidentified gene cassettes, which seemed likely to encode metallophosphatases (Ghodge and Raushel, 2018). A gene cassette was found to exhibit 72% amino acid identity with NikR, a transcription factor that regulates nickel uptake (Zeng and Zamble, 2017). In addition, we found some gene cassettes related to the type IV secretion system, type II system, their subunits, and secretion proteins to share a homology of 73% within the database and 43% with the N terminal region of pilus formation protein. These secretion systems are responsible for attachment, motility, colonization, and HGT (Sgro et al., 2018). This study provides a baseline for further studies on different varieties of biochar, in different environments, and at a different level to investigate their effects on integrons and their antimicrobial resistance.

## Conclusion

Integrons are genetic elements that acquire, rearrange, and express a diverse array of gene cassettes, including antimicrobial resistance genes in the bacterial community. Our study showed that the addition of biochar in the paddy soils could mitigate the integrons and integron-related AMR, affect the gene cassettes, and reduce the cassette diversity, both alone and in combination with manure. We have identified some novel gene cassettes of different functions. Integrons are important AMR indicators. Therefore, the application of sole biochar or in combination with organic manure is an alternative approach and the conversion of manure to biochar could serve as an approach for mitigating the spread of integrons and AMR genes, in the environment and possibly from soil to food and water reservoirs. The study provides a baseline for further studies on different varieties of biochar.

## Data Availability Statement

The original contributions presented in the study are included in the article/supplementary material, further inquiries can be directed to the corresponding authors.

## Author Contributions

NA, LJ, RW, and BH designed the study. NA, YL, and IzA processed the data and completed the study. NA, BH, and RW edited the manuscript. NA, RW, IsA, and KA revised the manuscript. All authors contributed to the article and approved the submitted version.

## Funding

This work was supported by the State Key Laboratory for Conservation and Utilization of Subtropical Agro-Bioresources (grant number SKLCUSA-a201913) and the project on the safe utilization of agricultural land.

## Conflict of Interest

The authors declare that the research was conducted in the absence of any commercial or financial relationships that could be construed as a potential conflict of interest.

## Publisher’s Note

All claims expressed in this article are solely those of the authors and do not necessarily represent those of their affiliated organizations, or those of the publisher, the editors and the reviewers. Any product that may be evaluated in this article, or claim that may be made by its manufacturer, is not guaranteed or endorsed by the publisher.

## Footnotes

^1^ https://www.ncbi.nlm.nih.gov/orffinder/
